# Site-Specific Incidence Rate of *Blastocystis hominis* and Its Association with Childhood Malnutrition: Findings from a Multi-Country Birth Cohort Study

**DOI:** 10.4269/ajtmh.22-0662

**Published:** 2023-04-10

**Authors:** Barbie Zaman Wahid, Md Ahshanul Haque, Md Amran Gazi, Shah Mohammad Fahim, Abu Syed Golam Faruque, Mustafa Mahfuz, Tahmeed Ahmed

**Affiliations:** 1 Nutrition and Clinical Services Division, International Centre for Diarrhoeal Disease Research, Bangladesh, Dhaka, Bangladesh;; 2 University of Nebraska-Lincoln, Lincoln, Nebraska

## Abstract

In this study, we investigated the potential association between the burden of asymptomatic *Blastocystis* spp. (*Blastocystis hominis*) infection and nutritional status among children under 2 years of age using the data collected from 1,715 children from eight distinct geographic locations, including Bangladesh, Brazil, India, Peru, Tanzania, Pakistan, Nepal, and South Africa. Childhood stunting, wasting, and underweight were the outcome variables, and *B. hominis* infection was the exposure variable of this present study. The presence of *B. hominis* in nondiarrheal stools was evaluated by TaqMan Array Cards. Site-specific incidence rates were estimated using Poisson regression, and multiple generalized estimating equation was used to assess the association between the *B. hominis* infection and nutritional status. The site-specific incidence rates of asymptomatic *B. hominis* infections per 100 child-months were higher in Tanzania, Peru, and South Africa when compared with the other study sites. Moreover, in terms of site-specific association, childhood stunting was significantly associated with asymptomatic *B. hominis* infection in Bangladesh (odds ratio [OR]: 1.62; 95% CI: 1.26–2.08), India (OR: 1.78; 95% CI: 1.46–2.16), Nepal (OR: 2.26; 95% CI: 1.60–3.21), Peru (OR: 1.47; 95% CI: 1.26–1.71), South Africa (OR: 1.57; 95% CI: 1.35–1.83), and Tanzania (OR: 2.46; 95% CI: 2.18–2.79) sites. Wasting was associated with *B. hominis* in the Brazil site only (OR: 3.19; 95% CI: 1.31–7.77). On the other hand, underweight was associated in the Bangladesh (OR: 1.89; 95% CI: 1.48–2.42), Brazil (OR: 4.41; 95% CI: 1.57–12.4), Nepal (OR: 2.25; 95% CI: 1.52–3.35), and Tanzania (OR: 1.68; 95% CI: 1.42–1.99) sites. Our analysis further reveals that the presence of additional pathogens may play a pathogenic role in children who have *B. hominis* infection.

## INTRODUCTION

*Blastocystis* spp. (*Blastocystis hominis*) is a widely distributed gastrointestinal protist that is commonly reported in tropical and subtropical countries.^1^ This protist is one of the few intestinal parasites with a prevalence of more than 5% in the general population of developed countries and up to 30–60% in developing countries.^2^ Based on epidemiological surveys, it is anticipated that this parasite has infected 1–2 billion individuals globally.^3^ According to recent microbiome studies, this protozoan parasite may colonize the human gut for long periods of time without causing symptoms, and *B. hominis* carriers have more bacterial diversity than non–*B. hominis* carriers, implying that it should be regarded as a commensal rather than a pathogen.^3^ Additionally, this organism has been associated with indicators of a healthy gut microbiota.^1^ However, it has also been associated with a decrease in protective bacteria in the feces, such as *Bifidobacterium* spp. and *Faecalibacterium prausnitzii*.^1^ Moreover, other researchers have attributed *B. hominis* to irritable bowel syndrome and inflammatory bowel disease as well as watery diarrhea, stomach pain, meteorism, a lack of appetite, and constipation.^2^ Furthermore, *B. hominis* may have a pathogenic role in immunocompromised patients.^2^^,^^4^^,^^5^ Studies have linked the parasite to cutaneous disorders and chronic or acute urticaria, in addition to atypical gastrointestinal symptoms.^2^ The organism causes no symptoms in most people who carry the parasite, but it can also be found in people who have diarrhea or other digestive issues.^1^ As a result, *B. hominis* can be an opportunistic organism that can have a negative impact on gut health when other pathogens are present.^1^ Hence, evidence of its pathogenicity in humans is speculative and inconclusive. There is insufficient evidence to determine if *B. hominis* causes disease or is beneficial to gut microbial diversity.^1^ Although there is still debate about its pathogenicity, recent research supports the pathogenicity of *B. hominis* in causing clinical symptoms in the absence of other intestinal infections.^6^

Children with recurrent diarrhea had a higher prevalence of intestinal parasitic infection, implying that parasitic infection should be investigated in cases of chronic or recurrent diarrhea.^6^ Moreover, intestinal parasite infections are most common in children of school age and may have a negative impact on growth.^7^ Similarly, prior literature revealed that a higher cumulative burden of diarrhea prior to the age of 24 months was linked to a higher prevalence of stunting at that age.^8^ In the absence of other pathogens, chronic diarrhea, nausea, anorexia, vomiting, dizziness, and flatulence caused by *B. hominis* have been found often in cases where the organism exists at higher numbers.^9^ This research backs up previous findings that *B. hominis* can cause diarrhea in children in underdeveloped nations.^9^ However, the precise role of *B. hominis* as a causative agent of growth faltering remains poorly understood. The findings of a study conducted among malnourished Bangladeshi adults imply that the presence of *B. hominis* in the human intestine has an impact on gut health and may play a pathogenic role in the presence of other pathogens.^1^ Hence, there could be a possible association of *B. hominis* with all forms of malnutrition among children from resource-constrained settings.

Therefore, we have structured this study with the purpose of investigating the site-specific incidence rates of *B. hominis* and its possible association with all forms of malnutrition among children aged 1–24 months.

## MATERIALS AND METHODS

### Study design and ethical statement.

The multi-country birth cohort Etiology, Risk Factors, and Interactions of Enteric Infections and Malnutrition and the Consequences for Child Health (MAL-ED) study, which was conducted across eight sites in South America, sub-Saharan Africa, and Asia (Bangladesh, Brazil, India, Nepal, Peru, Pakistan, South Africa, and Tanzania), has been previously described.^10^ In summary, from November 2009 to February 2012, 1,715 children were registered from the community within 17 days of birth at all eight sites and observed for up to 24 months. Each of the eight study sites' institutional review boards gave approval to the study.^10^ Every child's parents or legal guardians provided written, informed, and voluntary consent. All study protocols were carried out in accordance with the ethical requirements set by the regulatory authorities at each study location.

### Collection of anthropometric, sociodemographic, and morbidity data.

In accordance with 2006 WHO standards for children, anthropometric indices of length-for-age z-score (LAZ), weight-for-age z-score (WAZ), and weight-for-length z-score (WLZ) were derived using data from monthly anthropometric measurements taken up to the age of 24 months, using standard scales (secaGmbH & Co. KG, Hamburg, Germany).^11^ Anthropometric data, as well as information on birth history, household demographics, and maternal factors, were obtained from study participants across all sites at the time of recruitment.^10^ During household visits, which were conducted twice weekly, a full description of any morbidity and child feeding practices was acquired.^12^

Starting at the age of 6 months, socioeconomic data were collected every 6 months. The WAMI (water, sanitation, hygiene, asset, maternal education, and income) index, which spans from 0 to 1, is a socioeconomic status index for households that was calculated thereafter.^13^ A greater WAMI suggested a higher socioeconomic class.^14^ The WHO criteria for better water and sanitation were followed,^15^ and drinking water treatment was defined as filtering, boiling, or adding bleach.^16^

### Outcome variables.

The outcome variables of the present study were stunting, wasting, and underweight. Stunting, also known as linear growth faltering, occurs when a child's LAZ falls below −2 SD, according to the WHO child growth standards (LAZ <−2). Weight-for-length z-score was less than −2 SD (WLZ <−2) for wasted children, and weight-for-age z-score was less than −2 SD (WAZ <−2) for underweight children. A study participant's stunting, wasting, or underweight indicated an event of malnutrition.

### Collection of stool samples and assessment of enteropathogens.

Nondiarrheal stool samples were obtained on a monthly basis after enrollment and up to the age of 24 months at all study locations. In this investigation, no diarrheal samples were analyzed. Upon collection of these stool samples, no fixative was added to the stool samples, and the unprocessed stool aliquots were stored in −80°C freezers before being analyzed in the laboratory.^17^^,^^18^ Using procedures described elsewhere, TaqMan array cards, a customized multiplex quantitative polymerase chain reaction using compartmentalized primer-probe assays, were used to detect a possible 29 pathogens from each of the samples.^19^^,^^20^ As stated previously, the quantification cycle value of 35 was set as a threshold for the analysis.^19^ In our study, we analyzed the occurrence of the 18S rRNA gene of *B. hominis*, and positive cases were checked for co-infection with other distinct pathogens, including *Campylobacter jejuni*, enteroaggregative *Escherichia coli* (EAEC), enterotoxigenic *E. coli* (ETEC), norovirus, atypical enteropathogenic *E. coli* (aEPEC), *Shigella*/enteroinvasive *E. coli* (EIEC), and *Cryptosporidium* spp.^21^

### Statistical analysis.

**Figure 1.**
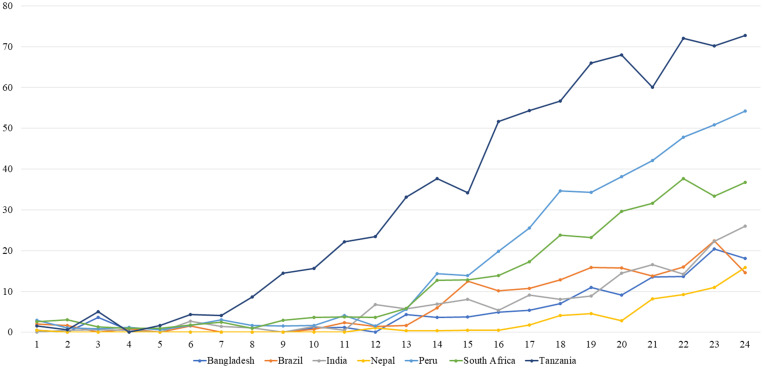
Site-specific prevalence of asymptomatic *Blastocystis* infection by children’s age in months.

Stata software (Release 14; StataCorp LP, College Station, TX) was used to analyze the data. Line graphs were used to illustrate the data in this study, including a line graph to assess the prevalence status of outcome variables with time and a variable for asymptomatic *B. hominis* infection (Figure 1). We used frequency and proportion for qualitative variables and mean and standard deviation for quantitative symmetric variables to summarize the data. Poisson regression was used to calculate the incidence rate in all of the study sites. The association between asymptomatic *B. hominis* infection and three forms of malnutrition (stunting, wasting, and underweight) was studied using multiple generalized estimating equations (GEEs) with binomial family, logit link function, and exchangeable correlation. The WAMI (water/sanitation, assets, maternal education, and income) index, child’s sex, maternal height, mothers’ having fewer than three living children, and site were adjusted in the multiple GEE model.^22^ Because of the birth cohort research, the child's age was used as a time variable in the model. Additionally, a *P* value of < 0.05 was used to portray significance, and the 95% CI was used to describe the findings.

## RESULTS

### General characteristics.

A total of 1,715 participants were enrolled throughout the eight study sites, and 34,622 monthly nondiarrheal stool samples were collected from the participants over the course of the study, commencing with enrolment after birth. The demographic characteristics of the study participants (*N* = 1,715) enrolled at each of the eight study locations are displayed in Table 1. Due to the unavailability of a subset of length measurements at the Pakistan site for this study, we eliminated children from that site (*N* = 246) for our analysis.

**Table 1:** General characteristics of MAL-ED study populations from November 2009 to February 2012 (*N* = 1,715)

Characteristics	Bangladesh	Brazil	India	Nepal	Peru	Pakistan	South Africa	Tanzania	Overall
Male sex	108 (51.4)	89 (53.9)	105 (46.3)	122 (53.7)	105 (54.1)	120 (48.8)	120 (50.6)	105 (50.2)	874 (51.0)
Birth weight, kg*	2.8 ± 0.4	3.4 ± 0.5	2.9 ± 0.4	3 ± 0.4	3.1 ± 0.4	2.7 ± 0.4	3.2 ± 0.5	3.2 ± 0.5	3.0 ± 0.5
Days of exclusive breastfeeding*	143.2 ± 42.7	93.7 ± 57.8	105.4 ± 42.9	92.5 ± 54.5	89.5 ± 61.3	19.9 ± 22.7	38.6 ± 26.3	62.2 ± 35	78.6 ± 57.7
WAZ at enrollment*	−1.3 ± 0.9	−0.2 ± 1	−1.3 ± 1	−0.9 ± 1	−0.6 ± 0.9	−1.4 ± 1	−0.4 ± 1	−0.1 ± 0.9	−0.8 ± 1.1
LAZ at enrollment*	−0.96 ± 1	−0.8 ± 1.1	−1 ± 1.1	−0.7 ± 1	−0.9 ± 1	−1.3 ± 1.1	−0.7 ± 1	−1 ± 1.1	−0.9 ± 1.1
LAZ at 24 months*	−2.0 ± 0.9	0 ± 1.1	−1.9 ± 1	−1.3 ± 0.9	−1.9 ± 0.9	N/A	−1.7 ± 1.1	−2.7 ± 1	−1.7 ± 1.2
Maternal age, years*	25.0 ± 5.0	25.4 ± 5.6	23.9 ± 4.2	26.6 ± 3.7	24.8 ± 6.3	28.1 ± 5.9	27 ± 7.2	29.1 ± 6.5	26.3 ± 5.9
Maternal weight, kg*	49.7 ± 8.5	62 ± 11.5	50.3 ± 9.3	56.2 ± 8.3	56.3 ± 9.6	50.7 ± 9.6	68 ± 15.3	55.7 ± 8.8	55.9 ± 12
Maternal height, cm*	149.0 ± 5.0	155.1 ± 6.7	151.1 ± 5.2	149.7 ± 5.3	150.2 ± 5.5	153.4 ± 5.7	158.7 ± 6.6	155.9 ± 5.9	152.9 ± 6.6
Maternal educational level < 6 years	133 (63.3)	22 (13.3)	80 (35.2)	59 (26)	44 (22.7)	202 (82.1)	5 (2.1)	75 (35.9)	620 (36.2)
Mother has < 3 living children	160 (76.2)	113 (68.5)	157 (69.8)	199 (87.7)	111 (57.2)	105 (42.7)	141 (59.5)	58 (27.8)	1044 (61)
Routine treatment of drinking water	130 (61.9)	10 (6.1)	7 (3.1)	98 (43.2)	32 (16.5)	0 (0)	12 (5.1)	12 (5.7)	301 (17.6)
Improved drinking water source	210 (100)	165 (100)	227 (100)	227 (100)	184 (94.9)	246 (100)	196 (82.7)	89 (42.6)	1544 (90.0)
Improved floor	204 (97.1)	165 (100)	222 (97.8)	109 (48)	69 (35.6)	81 (32.9)	231 (97.5)	13 (6.2)	1094 (63.8)
Improved latrine	210 (100)	165 (100)	121 (53.3)	227 (100)	66 (34)	197 (80.1)	232 (97.9)	19 (9.1)	1237 (72.1)
Monthly income < $150	69 (32.9)	161 (97.6)	19 (8.4)	106 (46.7)	58 (29.9)	115 (46.8)	179 (75.5)	0 (0)	707 (41.2)

LAZ = length-for-age z-score; MAL-ED = Etiology, Risk Factors, and Interactions of Enteric Infections and Malnutrition and the Consequences for Child Health; WAZ = weight-for-age z-score.

* Mean ± SD.

### Incidence rate of *B. hominis* infection.

The overall incidence rate (IR) of asymptomatic *B. hominis* infections per 100 child-months was 11.87 (95% CI: 11.47–12.28). The site-specific IRs per 100 child-months were: Bangladesh, 4.74 (95% CI: 4.11–5.48); Brazil, 6.99 (95% CI: 6.04–8.08); India, 6.71 (95% CI: 5.99–7.53); Nepal, 2.52 (95% CI: 2.11–3.02); Peru, 17.02 (95% CI: 15.75–18.38); South Africa, 13.04 (95% CI: 12.03–14.14); and Tanzania, 33.65 (95% CI: 31.84–35.55) (Table 2). The overall IR of *B. hominis* infection was 11.87% across all study locations. Tanzania had the highest IR of *B. hominis* infection per 100 child-months (33.65%), while Nepal had the lowest (2.52%).

**Table 2:** Site-specific incidence rate of asymptomatic *Blastocystis hominis* infections per 100 child-months

Sites	Incidence rate per 100 child-months (95% CI)
Overall	11.87 (11.47–12.28)
Bangladesh	4.74 (4.11–5.48)
Brazil	6.99 (6.04–8.08)
India	6.71 (5.99–7.53)
Nepal	2.52 (2.11–3.02)
Peru	17.02 (15.75–18.38)
South Africa	13.04 (12.03–14.14)
Tanzania	33.65 (31.84–35.55)

Incidence rates (95% CI) were calculated with the Poisson null model.

### Associations between *B. hominis* and other pathogenic enteropathogens.

Table 3 reflects the strength of associations between *B. hominis* and other pathogens. Co-infection were estimated using GEE, with *B. hominis* as the dependent variable and *Campylobacter*,* Giardia*, norovirus, ETEC, EAEC, aEPEC, tEPEC, *Shigella*/EIEC, and *Cryptosporidium* spp. as independent variables. *Blastocystis hominis* was positively associated with *C. jejuni/E. coli* (odds ratio [OR]: 1.12; 95% CI: 1.00–1.24; *P* = 0.040), *Giardia* spp. (OR: 4.62; 95% CI: 4.21–5.06; *P* < 0.001), ETEC (OR: 1.31; 95% CI: 1.20–1.44; *P* < 0.001), aEPEC (OR: 1.14; 95% CI: 1.04–1.25; *P* = 0.007), tEPEC (OR: 1.09; 95% CI: 0.96–1.23; *P* = 0.198), *Shigella*/EIEC (OR: 1.21; 95% CI: 1.08–1.36; *P* = 0.001), and *Cryptosporidium* spp. (OR: 1.47; 95% CI: 1.30–1.66; *P* < 0.001) infection. Norovirus (OR: 0.78; 95% CI: 0.69–0.88; *P* < 0.001) and EAEC (OR: 0.65; 95% CI: 0.59–0.70; *P* < 0.001) infections, on the other hand, were negatively associated with *B. hominis* infection. Table 2 summarizes the strength of the co-infection associations between *B. hominis* and other pathogens.

**Table 3:** The strength of associations between *Blastocystis hominis* and other pathogen as co-infection were estimated using GEE, where the dependent variable was *B. hominis* and independent variables were *Campylobacter jejuni*,* Giardia* spp., norovirus, ETEC, EAEC, aEPEC, tEPEC, *Shigella*, *Cryptosporidium* spp.

Co-infection	Unadjusted OR (95% CI)	*P* value	Adjusted* OR (95% CI)	*P* value
*Campylobacter jejuni*/*Escherichia coli*	1.29 (1.18–1.40)	< 0.001	1.12 (1.00–1.24)	0.040
*Giardia* spp.	4.89 (4.48–5.33)	< 0.001	4.62 (4.21–5.06)	< 0.001
Norovirus	0.78 (0.70–0.87)	< 0.001	0.78 (0.69–0.88)	< 0.001
ETEC	1.51 (1.40–1.63)	< 0.001	1.31 (1.20–1.44)	< 0.001
EAEC	0.74 (0.69–0.80)	< 0.001	0.65 (0.59–0.70)	< 0.001
aEPEC	1.16 (1.08–1.25)	< 0.001	1.14 (1.04–1.25)	0.007
tEPEC	1.08 (0.97–1.20)	0.160	1.09 (0.96–1.23)	0.198
*Shigella*/EIEC	1.53 (1.38–1.70)	< 0.001	1.21 (1.08–1.36)	< 0.001
*Cryptosporidium* spp.	1.88 (1.68–2.09)	< 0.001	1.47 (1.30–1.66)	< 0.001

aEPEC = atypical enteropathogenic *E. coli*; EAEC = enteroaggregative *E. coli*; EIEC = enteroinvasive *E. coli*; ETEC = enterotoxigenic *E. coli*; GEE = generalized estimating equations; OR = odds ratio; tEPEC = typical enterotoxigenic *E. coli*.

* Adjusted for sex; WAMI (water, sanitation, hygiene, asset, maternal education, and income) index, maternal height, and mother having fewer than three living children.

### Association between *B. hominis* infection and all forms of childhood malnutrition.

Table 4 shows the results of a separate analysis involving the association between the burden of *B. hominis* infection with all forms of malnutrition (stunting, wasting, and underweight) during 1–24 months of age. After adjusting for the relevant covariates, the overall infection of asymptomatic *B. hominis* infection over the study period across all study sites, excluding Pakistan, was found to have a significant association with stunting (OR: 1.91; 95% CI: 1.79–2.05; *P* < 0.001) and underweight (OR: 1.41; 95% CI: 1.29–1.54; *P* < 0.001); however, no association was observed with wasting (OR: 0.94; 95% CI: 0.77–1.15; *P* = 0.556).

**Table 4:** The site-specific strength of associations between the burden of *Blastocystis hominis* infection and all forms of nutritional status of the study participants

Form of malnutrition	Unadjusted OR (95% CI)	*P* value	Adjusted OR (95% CI)	*P* value
Stunting				
Overall	1.92 (1.81–2.04)	< 0.001	1.91 (1.79–2.05)	< 0.001
BG	1.80 (1.45–2.24)	< 0.001	1.62 (1.26–2.08)	< 0.001
BR	1.92 (1.09–3.40)	0.025	1.94 (0.91–4.11)	0.084
IN	1.71 (1.44–2.03)	< 0.001	1.78 (1.46–2.16)	< 0.001
NP	2.19 (1.61–2.96)	< 0.001	2.26 (1.60–3.21)	< 0.001
PE	1.52 (1.33–1.74)	< 0.001	1.47 (1.26–1.71)	< 0.001
SA	1.61 (1.40–1.84)	< 0.001	1.57 (1.35–1.83)	< 0.001
TZ	2.53 (2.27–2.83)	< 0.001	2.46 (2.18–2.79)	< 0.001
Wasting				
Overall	0.98 (0.80–1.19)	0.804	0.94 (0.77–1.15)	0.556
BG	1.39 (0.91–2.12)	0.125	1.29 (0.84–1.99)	0.250
BR	2.79 (1.39–5.57)	0.004	3.19 (1.31–7.77)	0.011
IN	0.93 (0.70–1.25)	0.634	0.88 (0.64–1.20)	0.422
NP	0.63 (0.19–2.07)	0.449	0.64 (0.19–2.13)	0.468
PE	1.06 (0.59–1.92)	0.849	1.10 (0.56–2.18)	0.785
SA	0.63 (0.34–1.18)	0.147	0.61 (0.33–1.15)	0.127
TZ	0.69 (0.35–1.36)	0.286	0.59 (0.29–1.19)	0.140
Underweight				
Overall	1.42 (1.31–1.53)	< 0.001	1.41 (1.29–1.54)	< 0.001
BG	2.06 (1.66–2.56)	< 0.001	1.89 (1.48–2.42)	< 0.001
BR	4.4 (1.55–12.44)	0.005	4.41 (1.57–12.4)	0.005
IN	1.08 (0.91–1.28)	0.385	1.06 (0.88–1.28)	0.527
NP	2.18 (1.56–3.05)	< 0.001	2.25 (1.52–3.35)	< 0.001
PE	1.08 (0.86–1.35)	0.530	1.06 (0.82–1.39)	0.642
SA	1.01 (0.81–1.26)	0.918	0.99 (0.78–1.27)	0.954
TZ	1.65 (1.41–1.93)	< 0.001	1.68 (1.42–1.99)	< 0.001

BG = Bangladesh; BR = Brazil; IN = India; NP = Nepal; PE = Peru; SA = South Africa; TZ = Tanzania. Adjusted in generalized estimating equation for sex; WAMI (water, sanitation, hygiene, asset, maternal education, and income) index, maternal height, mother having fewer than three living children and as co-pathogen infection, and site for overall estimates. Dependent variable: stunting (length-for-age z-score < −2 SD), wasting (weight-for-length z-score < −2 SD), and underweight (weight-for-age z-score < −2 SD). Independent variables: *B. hominis* infection.

When we looked at site-specific associations, stunting was found to be associated positively with the burden *B. hominis* infection over the study period across sites, including Bangladesh (OR: 1.62; 95% CI: 1.26–2.08; *P* < 0.001), India (OR: 1.78; 95% CI: 1.46–2.16; *P* < 0.001), Nepal (OR: 2.26; 95% CI: 1.60–3.21; *P* < 0.001), Peru (OR: 1.47; 95% CI: 1.26–1.71; *P* < 0.001), South Africa (OR: 1.57; 95% CI: 1.35–1.83; *P* < 0.001), and Tanzania (OR: 2.46; 95% CI: 2.18–2.79; *P* < 0.001). However, no such association was found in Brazil (OR: 1.94; 95% CI: 0.91–4.11; *P* = 0.084).

On the other hand, burden of *B. hominis* was found to be positively associated with wasting only in Brazil (OR: 3.19; 95% CI: 1.31–7.77; *P* = 0.011). Consequently, the burden of asymptomatic *B. hominis* infection was significantly associated with underweight in Bangladesh (OR: 1.89; 95% CI: 1.48–2.42; *P* < 0.001), Brazil (OR: 4.41; 95% CI: 1.57–12.4; *P* = 0.005), Nepal (OR: 2.25; 95% CI: 1.52–3.35; *P* < 0.001), and Tanzania (OR: 1.68; 95% CI: 1.42–1.99; *P* < 0.001). Table 4 depicts the site-specific strength of associations between the burden of *B. hominis* infection and all forms of nutritional status of the study participants.

## DISCUSSION

**Figure 2.**
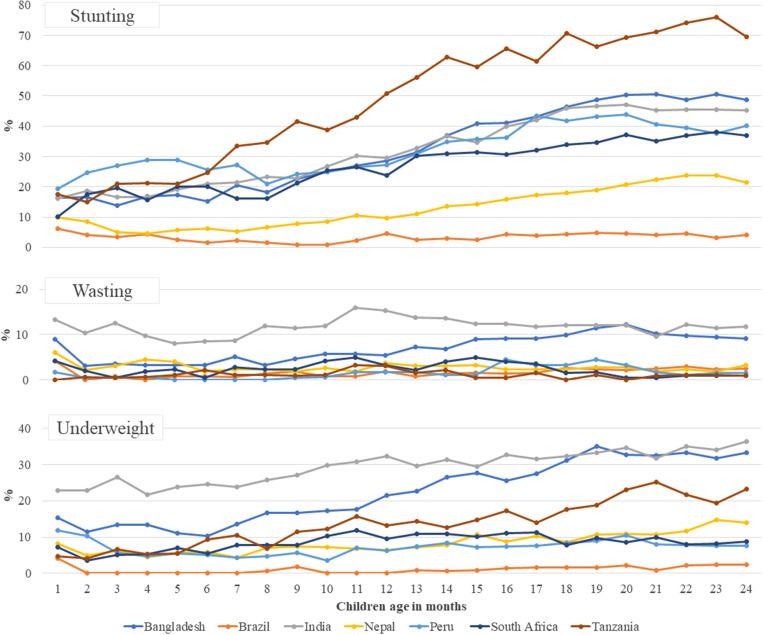
Site-specific prevalence of stunting, wasting, and underweight by children’s age in months.

According to epidemiological data, *B. hominis* is highly prevalent in tropical and subtropical settings, particularly in developing countries with inadequate hygiene conditions and consumption of contaminated food or water. Although its pathogenic potential is still questioned, *B. hominis* is commonly recognized as a pathogenic contributor to diarrhea, abdominal pain, and irritable bowel syndrome in people. In particular, in immunocompromised patients, such as those with HIV/AIDS or cancer, *B. hominis* infections can cause noticeable diarrhea.^23^^,^^24^ In this current literature, the association between asymptomatic *B. hominis* infection and various forms of childhood malnutrition (i.e., stunting, wasting, and underweight) in children enrolled in the MAL-ED birth cohort study were analyzed. Our data demonstrated a considerable discrepancy in the prevalence of asymptomatic *B. hominis* infections when compared with the other study sites, with incidence rates per 100 child-months being higher in Tanzania, Peru, and South Africa (Figure 2). Further observations into our site-specific assessment revealed a significant association between asymptomatic *B. hominis* infection and childhood stunting in Bangladesh, India, Nepal, Peru, South Africa, and Tanzania. To the best of our knowledge, this is the first study to investigate the associations between the protist *B. hominis* and all forms of malnutrition in children from birth to 2 years of age.

When compared with the other study sites, our findings revealed a significant disparity in the prevalence of asymptomatic *B. hominis* infections, with incidence rates per 100 child-months being higher in Tanzania, Peru, and South Africa. These findings corroborate previous findings from epidemiological data that *B. hominis* is highly abundant in tropical and subtropical settings, particularly in developing countries with poor personal hygiene, inadequate hygiene conditions, and consumption of contaminated food or water.^1^^,^^23^^,^^24^ According to our findings, the greatest incidence rate of *B. hominis* infection in children was reported in Tanzania (34%). The incidence of *B. hominis* found in this study is consistent with previous MAL-ED research on other enteropathogens, such as enterotoxigenic *Bacteroides fragilis*, enteropathogenic *E. coli*, and enteroaggregative* E. coli*, which found that the highest incidence rates were also found in Tanzania.^25^^–^^27^ Another study revealed a seasonal effect on *B. hominis* prevalence, which rose to 23.2% in the summer, compared with 13.7% in the winter.^28^ Summer water-based recreational activities may also be involved because human fecal contamination has been linked to *B. hominis* loads in recreational rivers, implying a higher risk of parasite infection in the summer.^28^ This seasonal pattern could explain *B. hominis* peaking in hot weather, and the prolonged summer season in Tanzania could be the underlying reason for the higher *B. hominis* occurrence in this particular study site.

Moreover, in our site-specific analysis, sites including Bangladesh, India, Nepal, Peru, South Africa, and Tanzania delineated a significant association between asymptomatic *B. hominis* infection and childhood stunting. According to a recent study, *B. hominis* infection can cause growth retardation, deterioration in cognitive and learning abilities, and a reduction in children's quality of life.^24^ Infection with *B. hominis* is becoming increasingly widely recognized as a severe threat to human health, and some *B. hominis* subtypes are pathogenic.^24^ Moreover, our result is consistent with a recent cross-sectional study of Australian aboriginal children aged 0–2 years that revealed an inverse relationship between *B. hominis* infection and height-for-age z-scores.^29^ Only Brazil depicted a significant association between *B. hominis* infection and wasting. On the other hand, a significant site-specific association between *B. hominis* and underweight was observed in Bangladesh, Brazil, Nepal, and Tanzania. Although there has not been a prior study tying the negative association between *B. hominis* infection and children’s WLZ, it can be interpreted that, in the presence of other pathogens, *B. hominis* damages the intestinal mucosa, resulting in reduced nutrient absorption and growth failure.^1^ A prior study conducted in Slovenia revealed the presence of *B. hominis* in fecal samples of diarrhea patients who had no other intestinal infections, suggesting an etiology that should be investigated further.^30^ Furthermore, when repeated stool examinations were obtained, it was discovered that 84% of individuals with *B. hominis* infection had at least one known pathogen other than *B. hominis*, including *Entamoeba histolytica*,* G. intestinalis*, and *D. fragilis*.^31^ Furthermore, following an increase in *B. hominis* detection rates in the 1990 s, investigations in the United States revealed *B. hominis* as a symptomatic mono-infection with characteristics similar to other amoeboid parasites, including *Cryptosporidium *spp. and *E. histolytica*.^32^ The behavior of *B. hominis* in people is comparable to that of *Giardia* spp. and *E. histolytica*.^32^

The findings also confirm that *B. hominis* meets all of the pathogenicity criteria set forth by *Giardia* spp. and *E. histolytica*, including the fulfillment of Koch's postulates.^32^ Another study identified a link between the presence of amoebic forms and *B. hominis* protease activity.^33^ The amoebic form of the protist *B. hominis* has previously been hypothesized to have a sticky surface that aids adherence to the intestinal epithelial cell lining.^33^ This adhesion could make it easier for amoebic forms to release proteases that degrade external membrane, similar to what was demonstrated in *E. histolytica*.^33^ Proteases have already been implicated as one of *E. histolytica*'s pathogenic factors.^33^ *Entamoeba histolytica* causes invasive intestinal disease that manifests as cramping, abdominal pain, and watery or bloody diarrhea over several weeks.^34^ Consequently, *E. histolytica*–associated diarrheal illness was found to be negatively associated with preschool children's linear growth in a study conducted in an urban slum in Mirpur, Dhaka.^35^ Therefore, it is probable that *B. hominis* is associated with diarrheal sickness and subsequent stunting in children.

*Blastocystis hominis* is disseminated via the fecal–oral pathway.^1^ Earlier studies have shown that unsanitary environmental factors, as well as crowded houses in developing countries, contribute to enteric pathogen transmission.^1^ Nonetheless, malnourished people have a higher chance of getting infected with enteropathogens. It is possible that a weakened immune system makes people more vulnerable to gastrointestinal infections.^1^ According to our data, the risk of infection with *B. hominis* is 12% higher in the presence of *C. jejuni*. A prior study found a significant association between *Campylobacter* spp. and *B. hominis*.^29^ This correlation revealed that the presence of *Campylobacter* spp. favors the presence of *B. hominis* and that the lack of one favors the absence of the other.^29^ Our study also delineated that the odds of infection with *B. hominis* were higher in the presence of pathogenic enteropathogens such as *Giardia* spp., *Cryptosporidium* spp., ETEC, and *Shigella*/Shiga toxin–producing* E. coli* (STEC) by 62%, 47%, 31%, and 21%, respectively. In Beni-Suef Governorate, Egypt, a prior study discovered a significant prevalence of intestinal parasite infection, particularly *Giardia–B. hominis* co-infection, in diarrheic young children (up to 12 years).^36^ This co-infection could be due to both parasites having the same environment, social conditions, transmission pattern, and age-prevalence curves.^36^ In another study, *G. intestinalis* was also reported to be the parasite most frequently associated with *B. hominis*.^36^ Furthermore, according to another study, largely asymptomatic *Giardia* spp. infections appeared to impair child growth, most likely due to malnutrition, while a similar link between *Giardia* spp. infection and nutritional status, as evaluated by anthropometric parameters in the Brazilian Amazon was recently discovered.^7^ This co-infection could also explain why *B. hominis* may be associated with impaired linear growth among children. Co-detection of *B. hominis* with *Cryptosporidium* spp. has also been observed in previous studies.^37^^,^^38^ Furthermore, *Cryptosporidium* spp. infection has been associated with stunting in numerous cross-sectional studies and a meta-analysis undertaken as part of the Global Burden of Disease Study.^39^ Furthermore, prospective studies on three continents (Africa, Asia, and South America) have shown that *Cryptosporidium* spp. infection in infancy and early childhood is a cause of stunting.^39^ We also observed an increase in the prevalence of *B. hominis* infection in the presence STEC, of another gut pathogen.^40^ Our analysis also exhibited that the odds of *B. hominis* infection are higher in the presence of ETEC, aEPEC, and tEPEC (organisms known for causing gut infections and altered intestinal health).^26^^,^^41^ The presence of the enteropathogenic *E. coli* (EPEC) genomic subtypes tEPEC and aEPEC was similarly inversely related to childhood linear growth in an earlier MAL-ED investigation.^26^ Conversely, in the presence of norovirus and EAEC, the risk of *B. hominis* infection decreases. This finding backs up prior claims that *B. hominis* is an opportunistic organism.^1^

### Limitation.

We have primarily evaluated the association between *B. hominis* and other pathogens in this study. The causality of this association, however, has not been investigated. Moreover, we have not measured sCD14 or any other marker of barrier dysfunction. Future research should thus concentrate on and take into account the aforementioned criteria to validate the association.

## CONCLUSION

When compared with other sites, we found that the incidence rate of asymptomatic *B. hominis* infections was greater in Tanzania, Peru, and South Africa. Furthermore, our site-specific analyses in Bangladesh, India, Nepal, Peru, South Africa, and Tanzania found a significant link between *B. hominis* and childhood stunting. In conclusion, findings from the present study imply that *B. hominis* has a potential pathogenic role in gut health and negatively affects growth development. Moreover, the pathogen appears to have harmful functions in the presence of certain enteric pathogens well known for gut infection. As a result, we urge the association of this ambivalent pathogen with growth failure as well as other potentially harmful enteropathogens to be investigated further, with a focus on the identification of *B. hominis* subtypes.

## Financial Disclosure

This work was supported, in whole or in part, by the Bill & Melinda Gates Foundation (Grant no. OPP47075). Under the grant conditions of the Foundation, a Creative Commons Attribution 4.0 Generic License has been assigned to the Author Accepted Manuscript version that might arise from this submission.
